# Comparative study of piroxicam and nitroglycerin on prostaglandin-modulated blood and electrolyte indices during di-estrous in female Wistar Rats

**DOI:** 10.5935/1518-0557.20220069

**Published:** 2023

**Authors:** Bernard O. Adele, Olayinka Oyinkansola Adebayo, Elsie Olufunke Adewoye

**Affiliations:** 1 Department of Physiology, Faculty of Basic Medical Sciences, University of Ibadan, Nigeria, West Africa

**Keywords:** dysmenorrhea, prostaglandins, piroxicam, nitroglycerin, blood and electrolyte indices

## Abstract

**Objective:**

Endogenous prostaglandins are involved in hemostasis, renal excretion of electrolytes, and implicated in dysmenorrhea. Piroxicam and Nitroglycerin are common drugs used in treating dysmenorrhea by inhibiting the cyclooxygenase pathway involved in prostaglandin production. However, studies comparing the effects of these drugs on prostaglandin-modulated hemostasis and renal function are lacking.

**Methods:**

Fifteen female rats (120-160g) were divided into 3 groups (20 per group), namely Control (distilled water, 0.3 mL), Piroxicam treated (3mg/kg) and Nitroglycerin treated (1 mg/kg). Di-estrous phase was confirmed in animals in each group using the Pipette smear method. Treatment was administered for 4 days covering the estrous cycle. Bleeding and clotting time were assessed and blood concentrations of sodium, potassium, urea and platelet counts were evaluated in all phases. Data were analyzed using one-way ANOVA and Newman-Keuls post-hoc test. Statistical significance was considered at *p*<0.0.

**Results:**

The nitroglycerin-treated group showed significant increases in blood potassium during di-estrous while the piroxicam-treated group showed significant increases in blood potassium, urea and clotting time with a significant decrease in sodium levels during di-estrous compared to controls. Results obtained in other phases were not significant compared to controls.

**Conclusions:**

The study showed that Nitroglycerin produces minimum alteration of blood and electrolyte indices compared to piroxicam during di-estrous.

## INTRODUCTION

Prostaglandins (PGs) are members of a group of lipid compounds derived enzymatically from fatty acids (Goodwin, 2010). They mediate many cellular functions such as cytoprotection of the gastric mucosa, renal function, gestation, parturition, neurotransmission, vasomotion, reproduction, metabolism, homeostasis, and are implicated in dysmenorrhea. Dysmenorrhea is the pain associated with menstruation and is classified as either primary or secondary based on the existence of underlying disease (Wong *et al*., 2009). Primary dysmenorrhea, the most frequent type of dysmenorrhea, is associated with elevated prostaglandin levels, uterine ischemia and painful menstrual cramps with no detectable pelvic pathology. In women clinically diagnosed with dysmenorrhea, endometrial PG levels were found to be significantly higher when compared with controls (Ostad *et al*., 2001). Reports include accounts of abdominal pain, cramps, headaches, and other systemic symptoms such as facial paleness, cold sweats, nausea, vomiting, and bloating in primary dysmenorrheic women (Deligeoroglou, 2000; Jia *et al*., 2006). The increased release of uterine PG produces significant degree of myometrial hyperactivity, which results in uterine hypoxia and ischemia associated with abnormal uterine contractions. Many non-steroidal anti-inflammatory drugs (NSAIDs) that are prostaglandin synthase inhibitors have been shown to be effective in the treatment of primary dysmenorrhea.

Piroxicam and nitroglycerin are common NSAIDs used in the management of dysmenorrhea (Wong *et al*., 2009; Marjoribanks *et al*., 2015). These drugs work by reducing the activity of cyclo-oxygenase pathways and thus inhibiting PG production (Burian & Geisslinger, 2005). Since prostaglandins are also involved in platelet plug formation, renal excretion of electrolytes, parturition and metabolism (Goodwin, 2010), the treatment of dysmenorrhea with NSAIDs is likely to disrupt these functional pathways. This study compared the effects of piroxicam and nitroglycerin on some prostaglandin-modulated blood and electrolyte indices during the di-estrous phase in female Wistar rats. The di-estrous phase of female Wistar rats was used as a model for dysmenorrhea because of the similarities it shares with the human menstrual phase.

## MATERIALS AND METHODS

### Materials

Drugs: Piroxicam (purchased under the brand name Feldene) and Nitroglycerin were obtained from Pfizer Pharmaceuticals, New York, USA.

### Experimental Animals and Grouping

Fifteen (15) female Wistar rats (120-160 g) were divided into three groups of 5 rats. The animals were acclimatized to laboratory conditions for 14 days prior to experimental procedures. They were housed in well aerated cages, maintained on standard rat pellets (Ladokun feeds, Nigeria) and allowed free access to drinking water. The guidelines of Animal Care and Use of the Research Ethics Committee, University of Ibadan, and of the National Research Committee, 1996 for the Care and Use of Laboratory Animals published by the National Academy Press, 2101 Constitution Ave. NW, Washington, DC 20055, USA, were followed.

Prior to the study, estrous cycle was established in each rat using the pipette smear method of Long & Evans (1922) and monitored for 12 days. The different estrous phases (pro-estrous, estrous, met-estrous and di-estrous) were established by the presence, absence or proportional numbers of epithelial cells (two types), cornified (keratinized) cells and leucocytes. Presence of predominance leukocytes in the vaginal smear was used to classify animals in the di-estrous phase (Long & Evans, 1922). Each group was then allocated 5 animals in the di-estrous phase as follows:

Group 1 served as controls and were given distilled water (0.3 mL).

Group 2 was treated with Piroxicam (3 mg/kg) (Sharp & Villano, 2012) and

Group 3 was treated with Nitroglycerin (1 mg/kg) (Sen *et al.,* 2011)

All treatments were administered orally for four days.

### Assessment of bleeding and clotting time

Bleeding time was assessed by manually amputating 5 mm of the rat tail tip. The time it takes bleeding to cease completely from the time of amputation is taken as the bleeding time (Dejana *et al*., 1982). Clotting time was assessed using blood samples withdrawn into capillary tubes from the retro-orbital sinus. Appearance of fibrin thread every thirty seconds was used as the endpoint (Dejana *et al*., 1982).

### Blood collection

Blood was collected daily from each rat through cardiac puncture under ether anesthesia into heparinized and plain bottles. Heparinized blood samples were subjected to platelet count evaluation (Harris *et al*., 1956).

### Biochemical assay

Blood concentrations of sodium, potassium and urea were evaluated using serum collected from plain blood samples after centrifugation at 3500rpm for 10 minutes. Levels of sodium, potassium and urea were determined using colorimetric assay kits (Randox Laboratories, UK).

### Statistical Analysis

Data were expressed as mean ± SEM and analyzed using one-way analysis of variance (ANOVA). Statistical significance was considered at *p*<0.05 using the New-man-Keuls post-hoc test.

## RESULTS

**Figure 1:**
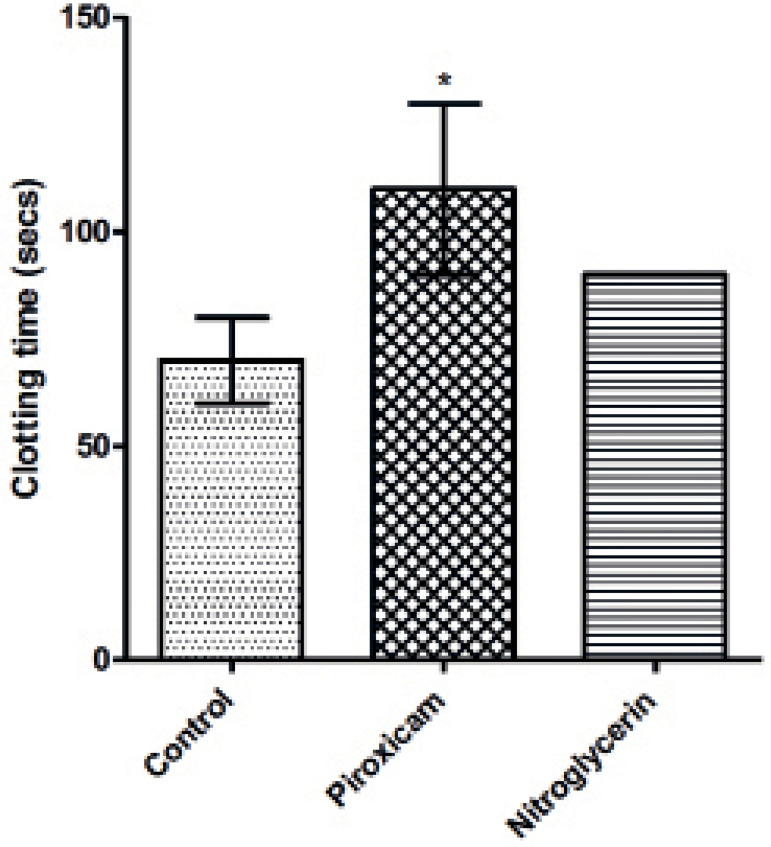
Clotting time in control and treated rats during di-estrous phase

The piroxicam-treated group showed a significant 57.14% (*p*=0.05) increase in clotting time compared to controls, which was 18.18% higher than the time seen in the nitroglycerin-treated group during di-estrous (Figure 1).

*Values are Mean ± SEM; p*≤0.05, n=5

*indicates values that are significantly different from controls.

**Figure 2:**
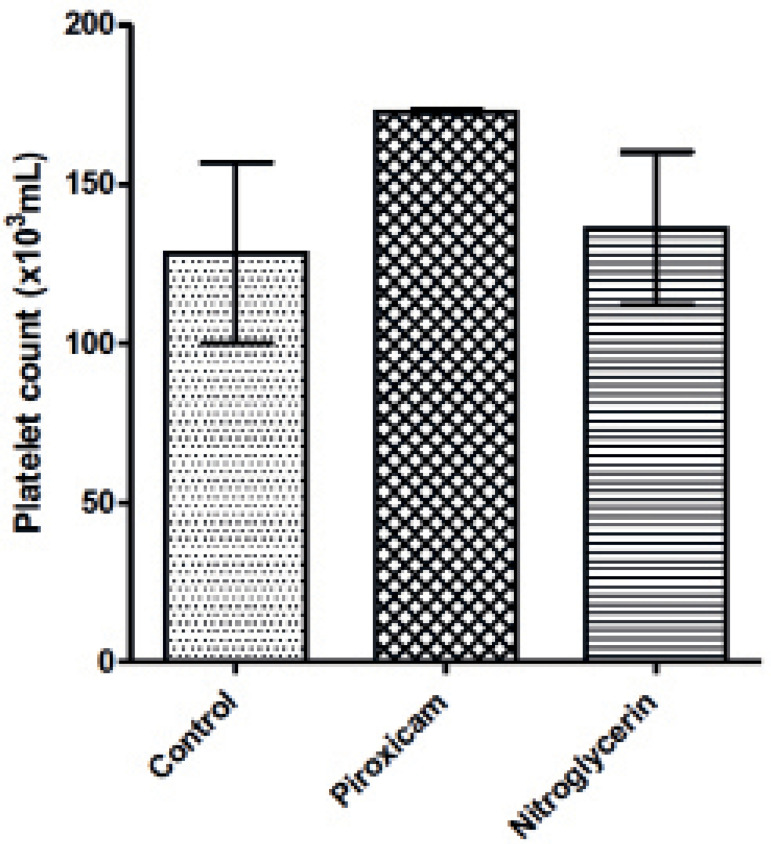
Platelet counts in control, piroxicam- and nitroglycerin-treated rats during di-estrous phase

Platelet count increased in the piroxicam-treated group when compared to controls and the nitroglycerin-treated group. However, this increase was not significant (Figure 2).

Values are Mean ± SEM; *p*≤0.05, n=5.

**Figure 3:**
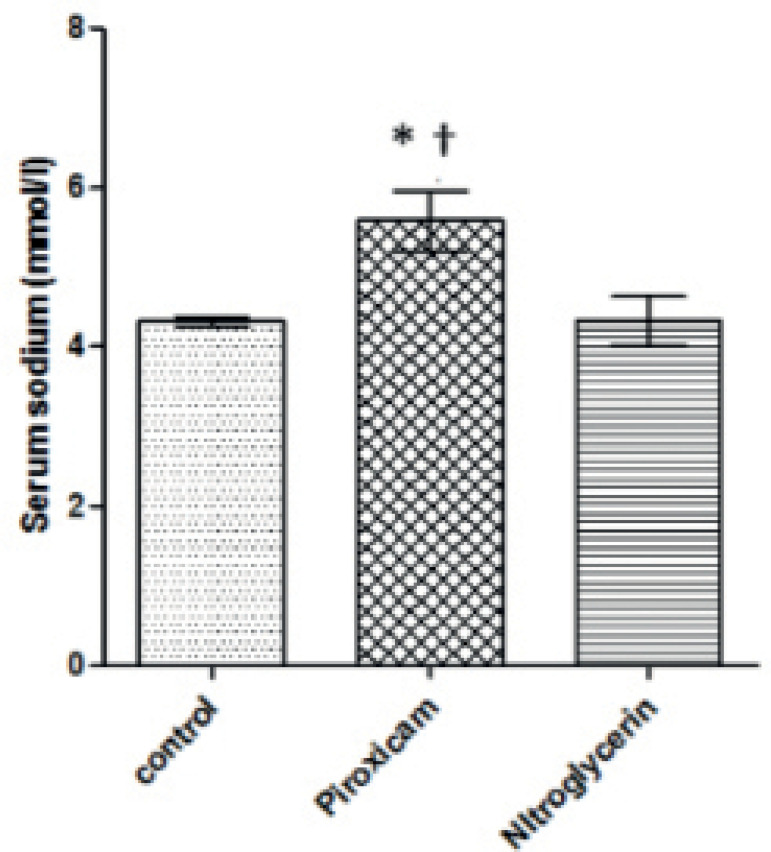
Blood sodium levels in control, piroxicam-and nitroglycerin-treated rats during di-estrous phase

**Figure 4:**
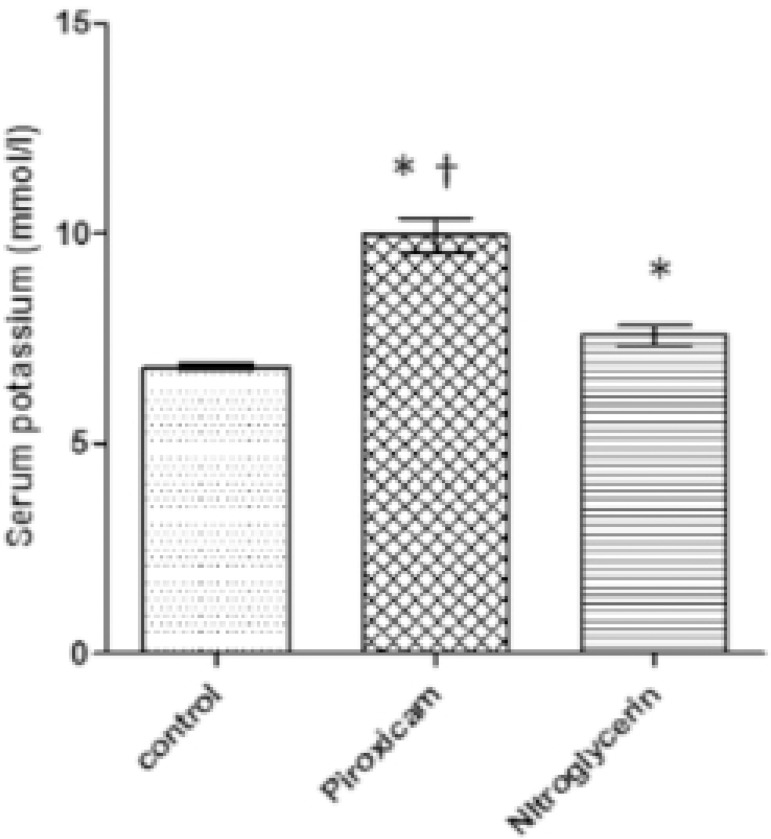
Blood potassium levels in control, piroxicam-and nitroglycerin-treated rats during di-estrous phase

**Figure 5:**
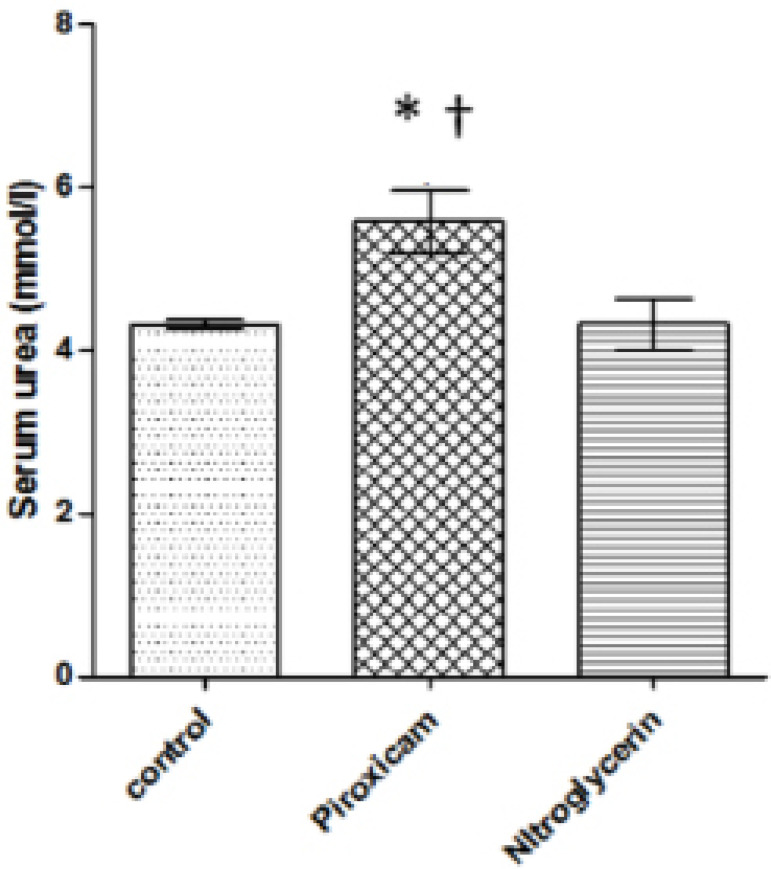
Blood urea levels in control, piroxicam-and nitroglycerin-treated rats during di-estrous phase

Nitroglycerin caused a significant 13.78% (*p*=0.05) increase in blood potassium levels when compared to controls, while piroxicam significantly (*p*=0.05) increased blood potassium (45.89%) and blood urea (29.17%) levels and significantly decreased (*p*=0.05) sodium levels (26.13%) compared to controls during di-estrous (Figures 3 to 5). In addition, the Piroxicam-treated group showed a significant (*p*=0.05) increase in potassium (24.02%) and urea levels (22.40%) and a significant 52.59% (*p*=0.05) decrease in sodium levels when compared to the nitroglycerin-treated group.

Values are Mean±SEM, *p*<0.05, n=5

*indicates values that are significantly different from control

^†^indicates significant difference from nitroglycerin.

Values are Mean±SEM, *p*<0.05, n=5

*indicates values that are significantly different from control

^†^indicates significant difference from nitroglycerin.

Results in other phases were not significant when compared to controls.

## DISCUSSION

Abnormal uterine contractions induced by PG production and release during the menstrual cycle are suppressed with the use of drugs that inhibit prostaglandin production (Wong *et al*., 2009; Marjoribanks *et al*., 2015). Suppression of this abnormal uterine activity enhances blood flow and reduces menstrual cramps and other symptoms that appear with dysmenorrhea. Results of this study showing increased levels of leukocytes during the di-estrous phase correlate with earlier reports of human menstrual phase characterized with endometrial leukocytes and increased myometrial prostaglandins (Vijayakumar & Walters, 1981; Salamonsen & Lathbury, 2000). It is therefore very possible that increased production and release of PGs reported during the human menstrual phase occurs during the di-estrous phase in female rats. Likewise, it is very possible that increased production and release of PGs accompanied with dysmenorrhea reported during the human menstrual phase also occurs during the di-estrous phase in female Wistar rats.

Commonly used NSAIDs such as piroxicam and nitroglycerin in managing dysmenorrheal pain potentiate their anti-nociceptive and analgesic effects by inhibiting the production of PGs and/or activating nitric oxide synthase (Bianchi & Panerai, 2002; Miclescu & Gordh, 2009). Prostaglandins, especially thromboxane A_2_ (TXA_2_), are involved in the promotion of platelet aggregation, vasoconstriction and smooth muscle cell proliferation during bleeding. Increased clotting time is an indicator of delayed platelet activation, which may have been due to the inhibition of TXA_2_ production (Tapiero *et al*., 2002). Increased clotting time recorded in piroxicam-treated rats may be due to the inhibitory effect of piroxicam on thromboxane A_2_ production (a kind of prostaglandin in the platelets) (Desouza *et al*., 1979). Increased clotting time is an indicator of delayed platelet activation (Dejana *et al*., 1982), and consequently may lead to hemorrhagic anemia in an individual when injured. On the contrary, platelet count increased with piroxicam treatment during di-estrous, although this increase did not result in reduced clotting time. Piroxicam may have prevented the activation of circulating platelets, thereby inhibiting the formation of clots or aggregates that might have helped shorten clotting time. This side effect was however not observed in animals treated with nitroglycerin.

Prostaglandins also play a significant role in renal control of sodium, potassium and blood urea nitrogen. In the kidney tubules, prostaglandins exert an inhibitory effect on vasopressin-modulated activities of adenylyl cyclase in collecting duct epithelial cells to attenuate vasopressin dependent transtubular water movement. Its inhibitory effects on cAMP in the thick ascending limb provide a tonic influence on Na-K-2Cl cotransporter expression (Fernández-Llama *et al*., 1999; Page *et al*., 2007). Removal of this inhibitory effect of PGs can inhibit water excretion and thereby promote the development of hyponatremia (Page *et al*., 2007). Treatment with piroxicam and nitroglycerin showed a hyperkalemic effect, while significant hyponatremic effect was recorded with piroxicam in rats at di-estrous as earlier reported by Kim (2008). The hyponatremic effect of piroxicam indicates that piroxicam inhibited the effects of PGs on sodium homeostasis. This hyponatremic effect suggests risks of renal impairment, metabolic disorders, muscle spasms, cardiac arrhythmia and nervous system disorders (Wharam *et al*., 2006; Kim, 2008). According to Kim & Joo (2007), potassium regulation is also influenced by the effect of PGs on renin secretion and angiotensin II-induced aldosterone secretion, among other factors. Lowering the effect of PGs on renin secretion and/or fall in aldosterone secretion can reduce potassium excretion and raise the extracellular levels of potassium. Increased extracellular potassium levels were observed in both piroxicam-treated and nitroglycerin-treated animals at di-estrous. Inhibitory activity of piroxicam on the COX2 pathway and stimulatory effects of nitroglycerin on Nitric Oxide (derived from iNOS) modulated proximal tubular Na-K-ATPase activity (Sharma, 2004) may have caused hyperkalemia. In addition to earlier reports linking use of NSAIDs to renal impairment (Kim, 2008; Bennett *et al*., 1996; Aprioku & Uche, 2013), this study also found elevated blood urea levels in piroxicam-treated animals in di-estrous. The positive effect of nitroglycerin on the renal excretion of metabolic waste might be attributed to its anti-oxidative activity (Sen *et al*., 2011).

The study shows that Nitroglycerin may produce minimal alteration of hemostasis and renal function during di-estrous compared to piroxicam.

## Figures and Tables

Values are Mean±SEM, *p*<0.05, n=5

*indicates values that are significantly different from controls

^†^indicates significant difference from nitroglycerin.
